# Acoustic stimulation effect on temporal processing skills in elderly subjects before and after hearing aid fitting

**DOI:** 10.1590/S1808-86942012000400004

**Published:** 2015-10-20

**Authors:** Maria Madalena Canina Pinheiro, Karin Ziliotto Dias, Liliane Desgualdo Pereira

**Affiliations:** PhD in Sciences - Graduate Program in Human Communication Disorders - Federal University of São Paulo; Adjunct Professor - Speech and Hearing Therapy Program of the Federal University of Santa Catarina; PhD in Sciences - Graduate Program in Human Communication Disorders - Federal University of São Paulo; Professor-collaborator in the Speech and Hearing Therapy Program of the Federal University of São Paulo. Chair of NESF - Speech and Hearing Therapy Study Group; Senior Associate Professor and PhD in Sciences - Graduate Program in Human Communication Disorders - Federal University of São Paulo; Senior Associate Professor of the Speech and Hearing Therapy Program of the Federal University of São Paulo); Federal University of São Paulo - Paulista Medical School

**Keywords:** auditory perception elderly, hearing, hearing tests

## Abstract

**Aging can alter, temporal** processing and affect speech perception.

**Aim**: To compare temporal processing auditory processing in elderly subject to and new hearing aid users.

**Materials and Methods**: The study included 60 elderly patients with bilateral sensorineural hearing loss. The procedures selected were the Duration Pattern Tests (DPT) and *gaps* in noise (GIN) test were used to analyze the responses of correct identification, and the temporal acuity threshold before and after the fitting of hearing aids. Study design: clinical and experimental research with non-probability sample of convenience.

**Results**: There was no statistically significant difference between the responses from GI and GII individuals. The elderly users of hearing aids had a lower *gap* detection threshold, greater recognition of *gaps* and of discrimination of the duration pattern in relation to when they were only potential users.

**Conclusion**: There was a deterioration in temporal processing skills, regardless of hearing loss degrees. Thus, the effect of acoustic stimulation by the use of a hearing aid improved resolution and temporal ordering.

## INTRODUCTION

Presbycusis is a type of hearing loss associated to aging, characterized by a descending bilateral sensorineural hearing loss, and it causes numerous problems both in communication and in the social lives of the elderly^1^.

Individuals with presbycusis have difficulties to discriminate acoustic clues - which help in speech understanding, especially in acoustically-challenged environments^2^, ^3^, ^4^. Besides the problems in the peripheral auditory system which arise from aging, the central nervous system auditory pathways are involved, causing difficulties in phoneme decoding, inter-hemisphere transmission and verbal and non-verbal stimuli coding^5^, ^6^, ^7^, ^8^. When aging affects the neurological processing of auditory information, the mental image of the acoustic event processed by the elderly will be of bad quality, with manifestations of auditory handicap.

Numerous papers bear evidence that among auditory processing skills, those associated with temporal processing are the most affected by aging^9^, ^10^, ^11^, ^12^, ^13^.

The temporal processing role in speech is the discrimination of subtle clues and similar words. Temporal processing involves temporal resolution aspects, temporal masking, temporal integration and sorting^14^.

The temporal resolution hearing skill is important for human speech understanding, being a prerequisite for reading^13^, ^15^. Human beings are capable of detecting *gaps* with 2 to 3 ms intervals presented in only one ear, and the auditory cortex neurons are responsible for detecting temporal acuity threshold^16^. Such skill involves the capacity to detect silence intervals among consecutive sounds,^9^, ^10^, ^17^ and it can be assessed by means of *gap* detection tests^5^, ^18^. The *gap* detection test in noise (GIN) has been recommended in current studies as a precursor tool to assess the temporal resolution auditory skill in children, young adults and elderly^18^, ^19^, ^20^, ^21^.

The temporal sorting auditory skill involves the participation of numerous perception and cognitive processes^22^, besides the stimulation of the right and left hemisphere and that of inter-hemispherical pathways, because the individual must, first, to recognize and discriminate two or more sounds in the order they occur in time and, afterwards, name the stimulus pattern^23^.

This skill must be analyzed by means of tests involving the temporal pattern recognition of pure tones, such as the Pattern Duration Test (PDT), which is considered a sensitive tool to identify lesions to the Central Nervous Auditory System (CANS), and it also does not suffer impacts from peripheral hearing loss^23^, ^24^.

The improvement in speech recognition caused by the use of a hearing aid usually happens between six and 12 weeks after using the amplification device, a time span called acclimatization^25^; however, changes may not happen in elderly with impairment in auditory processing hearing skills, especially that of temporal aspects associated to sound stimulus frequency, intensity and duration^26^.

The use of digital technology and the progress in signal processing have brought about great benefits concerning the communication of patients with presbycusis. Directional microphones, noise suppressors and non-linear amplification improve considerably the signal intelligibility in the presence of competitive noises^27^. Nonetheless, there is still very little evidence concerning improvements in auditory skills in temporal aspects by exposure to sound amplification.

With this study we hope to broaden the current knowledge vis-à -vis the effects of acclimatization in the neurological process of hearing, by means of behavioral tests of the auditory processing in elderly who are candidates to a hearing aid fitting.

Thus, the goal of the present study was to check and compare temporal processing responses in elderly candidates to hearing aid fitting, besides analyzing whether schooling and age impacted temporal processing skills.

## MATERIALS AND METHODS

This study is an experimental research with a non-probabilistic sample by convenience.

This study was approved by the Ethics in Research with Human Beings Committee of the institution with approval CEP number 1953/08. All the individuals included in the study signed an informed consent form authorizing their participation in the study.

The subjects of this study were selected among the elderly patients seen at NIAPEA - candidates to selection and fitting of a hearing aid, according to criteria from the Auditory Health-Care ordinance # 587, from 10/07/2004^28^.

We considered the elderly with more than 60 years of age, based on the National Elderly Ordinance^29^, which advocates this age range as the beginning of an age when one would be considered an elderly.

The initial sample was made up of 65 elderly in the age range between 61 and 85 years. During the assessments, two patients were taken off the sample because on the day of the assessment they had air conduction changes and decided against participating in the study because of difficulties in undergoing longitudinal assessments. The final sample was made up of 60 elderly, 20 men and 40 women. The individuals were gathered in two groups, called Group I (GI) and Group II (GII), based on the degree of mean values of the 500 and 4000 Hz sound frequencies in the audiogram. GI individuals had mean values between 41 and 50 dBHL in the frequency range between 500 and 4,000 Hz and, in GII, the mean varied between 51 and 70 dBHL.

The individuals included in the sample had to have the following criteria: no evidence of neurological impairment to prevent the understanding of the requested tasks, be native Brazilian Portuguese speakers, have symmetrical bilateral sensorineural hearing loss with hearing thresholds between 41 and 70 dBHL in the frequency range between 500 and 4,000 Hz, bilateral type A tympanometric curves^30^ and be a new user of intra-aural hearing aids without any prior hearing aid use experience.

The two participant groups were assessed with the procedures selected for this study at two different times. The first was before fitting the hearing aid, called First Assessment. The second time was after an effective time of hearing aid use, called Reassessment. In order to guarantee that the hearing aid was being used, follow up visits were scheduled between the First Assessment and the Reassessment, with one professional from the institution being responsible for checking the hearing aid fitting in these patients.

The temporal processing assessment was based on a Pattern Duration Test (PDT) and *Gap* in Noise Detection test (GIN). The temporal tests were employed at two different times, in the first assessment, before hearing aid selection and fitting, and, in the second, after a minimum of three months using the hearing aids.

The function measured with the PDT^23^ is the discrimination of sound patterns. The test stimulus is based on three 1,000 Hz tones, made up of 30 sequences, each sequence having three tones with different tone duration. The tone frequency is maintained at 1 KHz, and the tone duration varies, one of 250 msec, called short, and another of 500 msec, called long. The interstimuli intervals were maintained at 300 msec between the sequential tones, and the rise-descent time was kept at 10 msec. The test sequences were presented at an intensity of 30 dBSL based on the auditory thresholds in the frequencies between 500 and 2,000 Hz in both ears, with TDH-39 earphones. The patients were instructed to repeat the three-tone sequence in the same order he/she heard it.

The GIN^31^ test aimed at establishing the percentage of *gap* detection and the temporal acuity threshold. The CD recording was presented through earphones, and the test sound presentation level was of 30 dBSL, based on the mean hearing thresholds in the frequencies of 500; 1,000 and 2,000 Hz. We used track two for the training and tracks three and four were used to assess the right and left ears, respectively. Each track had six-second white-noise stimuli, with five-second intervals between the stimuli. The *gaps* are inserted in the white noise in given positions and with different durations, which can be 2, 3, 4, 5, 6, 8, 10, 12, 15 or 20 ms. With such procedure it was possible to assess the capacity to detect and discriminate mild differences between acoustic signals, in other words, the auditory skill of temporal resolution. We used the term “recognition” in the number of times the participants showed having identified the stimulus. The number of times the stimulus was detected was expressed in percentage and was called *“gap* recognition percentage”. The minimum value at which the individual perceived the *gap* in at least 4 of the 6 stimuli presented was called “temporal acuity threshold”. The normality criteria followed for this test were in accordance with Dias^21^. We must stress that the individuals who had GIN_Li greater than 20 ms were represented by a threshold of 22 ms for acoustic treatment purposes.

Before test reassessment, we utilized a tool available in the hearing aid fitting software called Data Logging. This tool enabled us to assess the mean number of hours of hearing aid use since the day of the first fitting. When the patient did not use the hearing aid effectively, the fitting problem was checked and a new follow up visit was scheduled. In such cases, the reassessment was only done after a minimum effective use time of 3 months. Upon reassessment, the patient was submitted to the tests using the hearing aid, and the transducer remained the TDH 39 earphone. The noise suppressor was turned off, so that it would not impact the responses during the GIN test.

The special auditory processing tests were presented from a Sony D-152 K Compact Disc player, coupled to a two-channel Grason-Stadler GS 61 Clinical Audiometer, with TDH 39 P earphones and MX-41 AR pad, calibrated according to the ANSI 69 standard.

In all the statistical tests utilized, we established a significance level of 0.05. The statistically significant values were marked with an asterisk [*] superscript. In case of a trend towards significant results, the calculated value was marked with the number symbol [^#^].

## RESULTS

The tests selected to assess temporal processing were employed before fitting the hearing aids and after a minimum period of three months of use and, a maximum of ten months. In the cases in which the reassessment was done in a period longer than three months, complications were looked for, such as technical issues with the hearing aid, ear wax preventing the insertion of the intra-aural hearing aid, difficulties fitting and handling the hearing aid. In the reassessment, there were three missing individuals, making up a total of 57 individuals.

On Table 1 we depict the values of the descriptive statistical mean values for the percentage of correct answers in the PDT by Assessment and Group.

**Table 1:** Descriptive statistics for the Percentage in PDT per Assessment and Group.

Assessment	Group	N	Mean	Standard Deviation	Minimum	Median	Maximum
First	GI	30	59.5	28.6	0	63.3	96.6
	GII	30	57.1	29.4	0	61.6	100
	Total	60	58.3	28.8	0	63.3	100
Re-assessment	GI	29	68.1	27.4	13.3	73.3	100
	GII	28	59.3	31.3	0	68.3	100
	Total	57	63.8	29.5	0	73.3	100

Covariance Analysis with Repeated Measures: G1 X G2 - p = 0.132. Difference between the % of the First Assessment X Reassessment - *p* = 0.013*. * statistically significant values.

We found a significant difference between the mean percentages of correct answers in the reassessment compared with that of the first assessment. The difference between the mean values of the two assessments was the same in both groups (*p =* 0.132). The estimate value of the difference in the two assessments was 5.7% (95% confidence interval: [2.1; 9.5].

In the first assessment, one individual from GI and two from GII did not discriminate any of the sequences which assessed the duration aspect between pure tones. In the reassessment, two GII individuals kept the same performance.

The GIN test was studied in relation to the percentage of *gap* recognition and as to the temporal acuity threshold in ms. In order to facilitate result presentation, the percentage of *gap* recognition was identified as GIN_%, and the temporal acuity threshold as GIN_Li.

The mean values from the descriptive statistics of the GIN test vis-à -vis the GIN_% are depicted on Tables 2 and 3.

**Table 2:** Descriptive analysis for GIN -Li per Ear, Assessment and Group.

Ear	Assessment	Group	N	Mean	Standard deviation	Minimum	Median	Maximum
Right	First	GI	30	29.7	16.5	0.0	31.6	65.0
		GII	30	27.7	19.5	0.0	27.5	65.0
		Total	60	28.7	17.9	0.0	30.0	65.0
	Re-assessment	GI	29	37.4	13.3	0.0	41.6	56.6
		GII	28	32.0	17.8	0.0	35.0	56.6
		Total	57¥	34.7	15.8	0.0	38.3	56.6
Left	First	GI	30	31.7	17.6	0.0	36.6	56.6
		GII	30	27.8	18.3	0.0	28.3	58.3
		Total	60	29.7	17.9	0.0	34.2	58.3
	Re-assessment	GI	29	38.1	14.9	0.0	41.6	56.6
		GII	28	28.7	16.6	0.0	30.0	55.0
		Total	57	33.5	16.3	0.0	38.3	56.6

Variance Analysis with Repeated Measures: First Assessment G1 X G2 - *p* = 0.362. Mean GIN_ % Assessment X Mean GIN_% Re-assessment - *p* < 0.001*. * statistically significant values.

¥ Three individuals did not come for behavioral tests reassessment.

**Table 3:** Descriptive statistics for GIN Li per Ear, Assessment and Group.

Ear	Assessment	Group	N	Mean	Standard deviation	Minimum	Median	Maximum
Right		GI	30	13.7	4.6	5	12	22
	First	GII	30	14.6	5.7	6	13.5	22
		Total	60	14.2	5.2	5	12	22
		GI	29	11.3	3.9	8	10	22
	Re-assessment	GII	28	13.3	5.2	6	12	22
		Total	57¥	12.3	4.6	6	10	22
Left		GI	30	13.1	5.2	8	10	22
	First	GII	30	15.2	5.6	6	15	22
		Total	60	14.1	5.5	6	12	22
		GI	29	10.9	4.3	8	10	22
	Re-assessment	GII	28	13.3	4.8	8	12	22
		Total	57¥	12.1	4.7	8	10	22

Variance Analysis with Repeated Measures: First Assessment G1 XG2- *p* = 0.263. Mean GIN_ % Assessment X Mean GIN_% Reassessment - *p* < 0.001*. * statistically significant values.

¥ Three individuals did not come for behavioral tests reassessment.

We found statistically significant differences between GIN_% mean values in the reassessment when compared to the first assessment. The increase happened in the *gaps* recognition percentage and it was the same for both groups. The mean difference between both assessments was 5.6% for the 95% Confidence Interval [3.2;8.1]. There were no differences between the *gap* recognition percentage mean values in noise in both groups (*p* = 0.362), and such result was valid for both assessments (*p* = 0.128).

**Figure 1:**
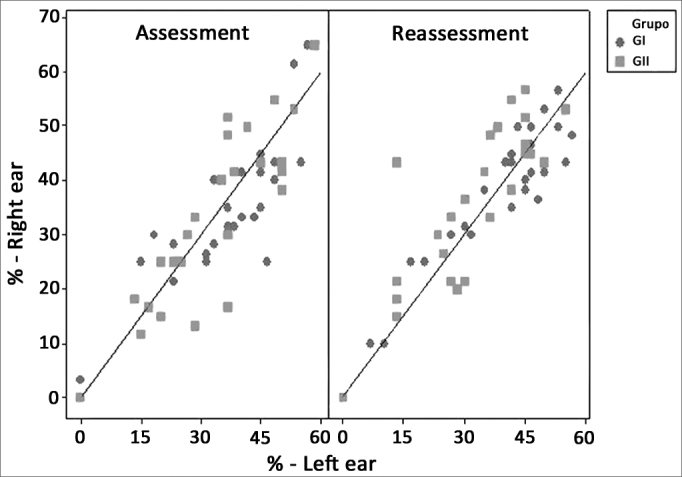
GIN_% scatter diagram according to the variable Ear and - GIN_% assessment- percentage value of *gap* recognition in the GIN test.

We assessed the agreement between the responses from the two ears, both in the first assessment and in the reassessment. The percentage scatter diagrams of correct answers in both ears and in the two assessments are presented on Figure 1.

We noticed that both in the GIN_Li as in the GIN_% there was a strong correlation between the two ears. The intraclass correlation coefficient for GIN_Li were 0.88 ([0.80;0.93] Confidence interval) in the assessment and 0.92 ([0.87;0.95] Confidence interval) in the reassessment. In the GIN_%, the intraclass correlation coefficient in the first assessment was 0.92 [0.86;0.95] and, in the reassessment it was 0.90 [0.84;0.94].

Table 3 depicts the GIN_Li (ms) per Ear, Assessment and Group. In the first assessment, six individuals from the GI and eight individuals from the GII did not recognize the *gap* in its maximum value in the test; as far as reassessment is concerned, only one individual from GI and four from GII did not identify the Maximum *gap* in the GIN *gap* that was 20 milliseconds.

There was a significant difference between the mean GIN_Li values in the two assessments, and the reassessment mean was lower than that in the first assessment. The mean drop in the threshold was -2.2 ms (95% confidence interval: of [-2.8;-1.5]), being equal in the two groups; there were no differences between the groups in the mean values of the temporal acuity threshold, and such result was valid for the two assessments (*p* = 0.373).

We noticed that in the GIN_Li there was an agreement between the two ears. The intraclass correlation coefficients were: 0,88 in the assessment (Confidence Interval [0.80;0.93]) and 0.92 in the reassessment (Confidence interval [0.87;0.95]).

Table 4 depicts the results of the association between the percentage of correct answers in the PDT and GIN with Age and Schooling.

**Table 4:** Spearman correlation coefficients from the temporal tests with Age and Schooling.

	Correlation Coefficients		
	PDT	GIN_%	GIN_Li
Age	r = -0.08 (*p* = 0.547)	r = -0.14 (*p* = 0.286)	r = 0.11 (*p* = 0.407)
Schooling	r = 0.55(*p* < 0.001*)	r = 0.21 (*p* = 104)	r = -0.25 (*p* = 0.058)

* statistically significant values.

We noticed a positive correlation between schooling and the percentage of correct answers in the PDT.

There was a trend towards a negative correlation between GIN_Li and schooling.

**Figure 2:**
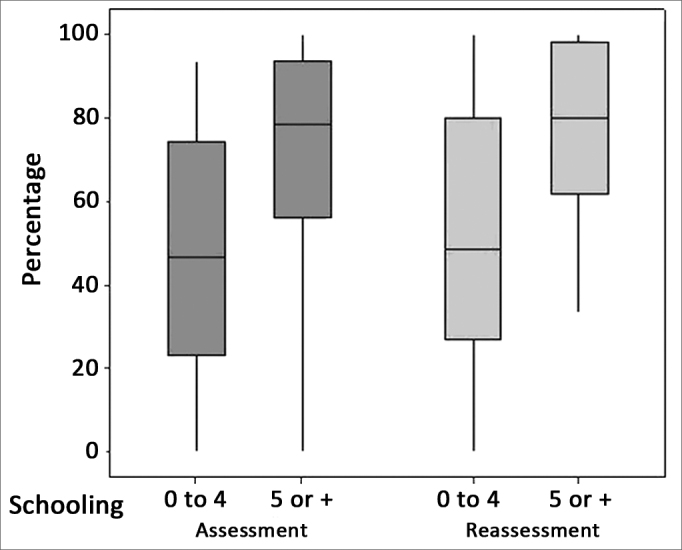
Distribution of individuals according to the percentage of correct answers in the PDT in both assessments according to schooling - PDT: Pattern Duration Test.

The distribution of correct answers of the two assessments and schooling ranges can be observed in Figure 2.

## DISCUSSION

The new users of hearing aid in this study were followed for a minimum period of 12 weeks of use in order to analyze the effects of acclimatization. This was all done based on the studies by Gatehouse^25^, who found an improvement in speech recognition after a minimum time of six and 12 weeks using the hearing aid.

In the present study, the degree of hearing loss did not influence performance in the PDT. G1 individuals found three sounds in rapid sequence similarly to G2 individuals (Table 1). We found a large result variability in each one of the groups.

Studies have shown that PDT performance in young adults and elderly populations with and without hearing loss was worse in elderly when compared to the young individuals in this task, without influence of mild to moderate hearing loss and with a worse performance in disorders of Central Nervous System and Brain auditory pathways^23^, ^32^, ^33^, ^34^, ^35^, ^36^. Studies in the specialized literature have shown that, among the elderly, the mean value of correct identification of a series of three brief tones in sequence (tonal PDT) varied between 43.75% and 69%^33^, ^35^, ^36^. Among adults^23^, ^32^, a better performance was found, with mean values of correct identifications higher than 83%.

Thus, the studies show that the aging process deteriorates the temporal ordering process^33^, ^34^, ^35^, ^36^ and the mild-to- -moderate cochlear hearing loss did impact performance in this task^13^, ^23^, ^33^.

In the present study, the moderate or moderately severe hearing loss did not impact the performance of the elderly in these tasks of recognizing the duration pattern, and the mean values are within the range of mean values of the studies with elderly listed in the literature^33^, ^34^, ^35^, ^36^.

Numerous studies report that the process of selecting and fitting hearing aids must consider the HLD (C) caused by the age effect, as the difficulty to discriminate temporal clues which identify speech contrasts^2^, ^3^, ^26^. There is still no consensus in the specialized literature whether only using a hearing aid improves auditory skills or if it is necessary to perform and auditory training so as to have a change in auditory behavior. Studies have indicated that the hearing aid enables improvements in auditory skills or whether it is necessary to perform auditory training so as to have changes in the auditory behavior. Studies indicate that the hearing aid brings about better acoustic information, notwithstanding it is not able to modify neural connections^26^, ^37^. Another study shows an improvement in temporal ordering auditory skills using only hearing aids with digital technology^34^.

In the present study, we noticed that after a period of daily use of the hearing aid, there was an improvement in recognizing the duration pattern both in GI (mean of 68.1%) and GII (mean of 59.3%), agreeing with a study published in the specialized literature^34^. The mean difference between the two PDT assessments was 5.7%.

In the GIN test (Tables 2 and 3), the degree of hearing loss did not impact the temporal acuity threshold results and *gap* recognition percentage in the GIN test.

National and international literature studies show that elderly have a lower percentage of *gap* recognition and a greater threshold of temporal acuity than children and youngsters^18^, ^19^, ^21^, ^35^, ^38^, ^39^, ^40^. In the studies involving elderly^21^, ^35^, ^39^, they noticed that *gap* recognition in noise varied from 39.1% to 57.6% and the acuity threshold varied between 7.3 and 10.2 ms. In children, youngsters and adults, the recognition threshold was higher than 70% and the temporal acuity threshold varied between 3.9 and 5 38 ms15,18,19,20,38,40

In the elderly with hearing loss, this threshold can be higher than in elderly without hearing loss^17^, ^35^. Notwithstanding, other papers did not report hearing loss influence in *gap* detection thresholds^4^, ^5^, ^13^, ^41^. Thus, we recommend that further studies are needed in order to check the effects of age and hearing loss in temporal resolution^15^.

In the present study, the mean percentage of *gap* recognition was lower and the temporal acuity threshold was higher than what was reported about elderly in the literature^21^, ^35^, ^39^. We noticed that there was no influence of the degree of hearing loss in the temporal resolution auditory skill (Tables 2 and 3), corroborating findings in the specialized literature^4^, ^5^, ^13^, ^41^.

Numerous papers state that there are no differences between the temporal acuity threshold and the percentage of *gap* recognition according to the variable Ear^15^, ^18^, ^19^, ^21^, ^35^, ^40^.

In the present study we noticed that both in the GIN_% and in the GIN_Li there was a strong agreement between the two ears (Figure 1), in agreement with the studies published in the specialized literature. We suggest the binaural employment of the test, since no differences were found between the ears.

One of the main complaints of elderly patients with hearing loss is the difficulty to understand speech in noise. Numerous studies showed that temporal resolution is affected by aging and causes difficulties to understand speech in noise^1^, ^27^, ^42^, ^43^. Studies show that the process of selecting and fitting hearing aids must not consider only the quantitative improvements in hearing thresholds, since many individuals did not show benefits brought about by changes to hearing skills associated with temporal processing^2^, ^37^, ^41^.

The inclusion of tests which assess the Central Auditory Processing in the process of selecting and fitting hearing aids has been increasingly recommended by authors in the specialized literature^37^.

In the present study, the percentage of *gap* recognition increased and the temporal acuity threshold reduced after a time using the hearing aid. We noticed that during the reassessment there was a statistically significant improvement in the temporal resolution hearing skill, and the temporal acuity threshold had a 2.2 ms reduction, and the percentage of *gaps* recognition in noise increased 5.6% both in the GI and in GII.

We must stress that in the first assessment, 14 individuals did not detect *gaps* in the noise range, being represented with the 22 ms threshold. Upon reassessment, only five individuals kept the 22 ms threshold. These findings suggest that there was an effect of the acoustic stimulation in the CAS information processing after the hearing aid fitting.

In the present study, the mean value of the GIN test temporal acuity threshold was high in the first assessment (mean of 14 ms), and in the reassessment (12 ms), and the *gap* recognition percentage was low (mean of 29%), compared with studies published in the specialized literature featuring young people^18^, ^19^, ^38^. By the same token, the PDT had a low recognition percentage of the duration pattern in the first assessment (mean 58.3%) and in the reassessment (mean 63.8%), compared to studies in young populations^23^, ^32^. Nonetheless, in the current study, there was no correlation between the temporal acuity threshold and the percentage of *gap* recognition with age, nor the recognition of duration pattern with age (Table 4). It is believed that aging causes changes in temporal ordering and resolution skills. Nonetheless, there was no correlation in the performance of elderly in the present study in temporal processing tests in the age range between 61 and 85 years.

We noticed that, in the sample investigated, there was a positive correlation between age and PDT performance, that is, the more years of schooling, the better the PDT performance (Table 4). Upon reassessment, we noticed that the low schooling individuals as well as those with high schooling had a better recognition of the duration pattern (Figure 2) with the use of a hearing aid. As far as the GIN is concerned, there is a trend concerning a negative correlation (*p* = 0.058) between the temporal acuity and schooling, that is, as schooling increases, there is reduction in the temporal acuity threshold.

Studies which assessed the temporal ordering and resolution skills in elderly with schooling higher than that in the present study, showed a better performance both in PDT as in the GIN_Li^21^, ^35^. We compared these data with the ones in the present study and we could infer that schooling influences the tasks which involve the participation of temporal ordering and resolution of hearing skills.

We believe that the application of the GIN and the PDT tests can be an important tool to help monitor the benefits of fitting a hearing aid in elderly patients.

## CONCLUSIONS

• Insofar as temporal processing/ordering skill and temporal resolution are concerned, there was a decline in skills, regardless of the hearing loss level, showing the effects of aging;
• There was an effect of the acoustic stimulation in the processing of information in the Central Nervous System, after acclimatization with the hearing aid, because the individuals had an improvement in their hearing skill, temporal ordering and temporal resolution;
• We found that schooling influenced resolution and temporal ordering tasks in the elderly.
